# Effect of FOXP2 transcription factor on immune infiltration of thyroid cancer and its potential clinical value

**DOI:** 10.3389/fimmu.2022.982812

**Published:** 2022-09-20

**Authors:** Lianghui Xu, Zheyu Yang, Qiwu Zhao, Haoran Feng, Jie Kuang, Zhuoran Liu, Linxie Chen, Lin Zhan, Jiqi Yan, Wei Cai, Weihua Qiu

**Affiliations:** Department of General Surgery, Ruijin Hospital, Shanghai Jiao Tong University School of Medicine, Shanghai, China

**Keywords:** FOXP2, thyroid cancer, immune infiltration, clinical prognosis, diagnosis

## Abstract

**Background:**

The clinical outcomes are not always favorable in certain thyroid cancer patients. The effect of Forkhead-box family on immune cells infiltration and tumor microenvironment in thyroid cancer was explored. The role of FOXP2 in tumor invasion and recurrence was investigated consequently.

**Methods:**

TIMER and GEPIA were firstly employed to compare FOXPs expression in normal and cancer tissues from multiple human cancers. The results from database were confirmed by quantitative Real Time-PCR and Western blot in matched thyroid cancer and adjacent normal tissues, in addition to a panel of thyroid cancer cell lines and normal thyroid cell. GEPIA platform was employed to discover the possibility of FOXPs as prognostic indicator. TISIBD and UACLCAN were then employed to estimate the influence of FOXPs on lymph node metastasis and tumor staging. GEPIA analysis was initially employed to analyze correlation of FOXPs and tumor immune infiltrating cells, and TIMER dataset was then included for standardization according to tumor purity.

**Result:**

Different member of FOXPs showed divergence in expression in various cancer tissues. Lower FOXP1, FOXP2 and higher FOXP3, FOXP4 levels could be identified in thyroid cancer tissues when compared with matched normal tissue. There was an inverse correlation between FOXP2, FOXP4 and immune invasion, whereas FOXP1 and FOXP3 were positively correlated. FOXPs showed remarkable correlations with multiply immune cells. More importantly, only FOXP2 showed the significant effect on recurrence and tumor staging.

**Conclusion:**

As immune regulatory factor, the reduction of FOXP2 may affect tumor microenvironments and immune cells infiltration, enhance tumor immune escape, and promote recurrence of thyroid cancer. FOXP2 could be a new potential diagnostic and prognostic marker.

## Introduction

Thyroid cancer (THCA) is the most common type of head and neck cancer, and the incidence is increasing worldwide (1–3). Although the majority of THCA patients have a good clinical prognosis after standardized therapies of surgery, radioactive iodine, and TSH inhibition, the clinical outcomes in certain patients are not always favorable (4, 5). Recent studies have confirmed that infiltration of different types of immune cells might be involved in neoplastic transformation of thyroid cancer, which suggested the role of the immune microenvironment in THCA (6).

Human Forkhead-box (FOX) family comprises diverse tissue and cell type-specific transcription factors with a conserved winged-helix DNA-binding domain (DBD) or forkhead domain, which constituted 19 subfamilies (A-S) (7). As transcriptional factors, FOXs play an important role in biological processes, including metabolism, development, differentiation, proliferation, apoptosis, migration, and invasion (8). Among subfamilies, FOXP, including FOXP1, FOXP2, FOXP3, and FOXP4, is closely associated with the genesis and the development of several malignancies (9). Dysregulation of FOXPs expression as the result of copy number alterations or chromosomal translocation was shown to be involved in development of various malignancies (9–11). Furthermore, FOXP were also shown to be essential for the development and maintenance of immunocyte (12). Therefore, recognizing the role of FOXP in the immune microenvironment and carcinogenesis of THCA will be helpful in the identification of new diagnostic, prognostic markers, and therapeutic interventions (13).

In present study, TIMER and GEPIA databases were employed to analyze the clinical significance of FOXPs. Then, the expression analysis of FOXPs in THCA were performed through multiple bioinformatics tools. The correlation between FOXPs expression and THCA patient prognosis was analyzed using the TCGA data. Evidenced by Western blot and Real Time-PCR were also used for further validation of our discovery. Furthermore, we validate these above outcomes *via* our own thyroid cancer clinical data. Subsequently, the correlation of immune cell infiltration and THCA were performed by TIMER and GEPIA. These findings demonstrate the potential clinical value of FOXPs in THCA. In particular, FOXP2 shows great potential in the diagnosis and may provide a new treatment strategy for THCA patients.

## Material and methods

### TIMER

TIMER (Tumor Immune Estimation Resource) is a comprehensive resource to systematically evaluate the clinical impact of different immune infiltrates across diverse cancer types (https://cistrome.Shinyapps.io/timer/). 10897 samples of 32 cancer types were obtained from the TCGA dataset and plotted on the TIMER platform to estimate the richness of immune infiltration. We used TIMER to analyze the expression of FOXPs in different human cancer. And the correlation between the expression of FOXPs and the abundance of immune infiltration. In addition, we also analyzed the marker genes of TIICs (tumor immune infiltrating cells) and studied the correlation between the expression of FOXPs and TIICs marker genes. Combined with the related role of TIICs, our selected TIICs markers include CD8+ T cells, T cells (general), B cells, monocytes, TAMs, M1 macrophages, M2 macrophages, neutrophils, natural killer (NK) cells, dendritic cells (DCs), T-helper 1 (Th1) cells, T-helper 2 (Th2) cells, follicular helper T (Tfh) cells, T-helper 17 (Th17) cells, Tregs, and exhausted T cells.

### GEPIA

GEPIA (Gene Expression Profiling Interactive Analysis), is a way of RNA sequencing analysis. We used GEPIA to analyze the expression difference between THCA and normal tissues. GEPIA was used to analyze the relationship between the expressions of FOXPs and the survival rate of THCA. Meanwhile gene markers of TIICs were analyzed to investigate the correlation between FOXPs expression and gene markers of TIICs *via* correlation modules.

### Cell culture and patient samples

Human thyroid cancer cell lines CAL-62, KHM-5M, BHT-101, B-CPAP, and normal thyroid cell lines Nthy-ori-3-1 were purchased from the China Center for Type Culture Collection (CCTCC, China). The CAL-62 and BHT-101 cells were cultured in DMEM medium supplemented with 10% and 20% fetal bovine serum (Gibco, USA) at 37°C in 5% CO2, respectively. KHM-5M, B-CPAP and Nthy-ori-3-1 were cultured in RPMI-1640 medium supplemented with 10% fetal bovine serum (Gibco, USA) at 37°C in 5% CO2. A total 15 of DTC patients who underwent radical thyroidectomy in Ruijin Hospital of Shanghai Jiaotong University Medical College and were confirmed as DTC by postoperative histopathological examination were included in this study. All samples were obtained with the patients’ informed consent, and the samples were histologically confirmed by at least 2 pathologists independently in a double-blinded fashion.

### RNA extraction and quantitative Real Time-PCR

Total RNA was isolated from fresh DTC tissues using TRIzol reagent (15596018, Invitrogen, USA) and then reverse transcribed into cDNA with gDNA Eraser using the HiScript III RT SuperMix (Vazyme, China) according to the manufacturer’s instructions. Quantitative PCR experiments were performed using ChamQ SYBR Color qPCR Master Mix (Vazyme, China). Briefly, this procedure included 60s of preincubation at 95°C and 40 cycles of denaturation at 95°C for 5s, annealing at 60°C for 15s, and extension at 72°C for 45s. The data was calculated using 2-ΔΔCt method.

### Western blotting

Western blotting was performed as previously described (14). Briefly, cells were solubilized in SDS-Sample Buffer containing 5% 2-Mercaptoethanol in the presence of ProtLytic Protease and Phosphatase Inhibitor Cocktail (New Cell & Molecular Biotech, China), 30-50 μg protein was separated by 10% or 12.5% SDS-PAGE gel and transferred to PVDF membranes. The membranes were blocked with 5% bovine serum albumin (BSA) for 2h and then were incubated at 4°C overnight with primary antibodies. The antibodies used in this study included anti-FoxP2 (Cell Signaling Technology, 5337), and anti-GAPDH (abcam, ab8245). Membranes were then incubated with secondary antibody [HRP-conjugated Affinipure Goat Anti-Mouse IgG (H+L) (Proteintech, SA00001-1), HRP-conjugated Affinipure Goat Anti-Rabbit IgG (H+L) (Proteintech, SA00001-2)] for 2h at room temperature and visualized using an enhanced chemiluminescence detection system (Tanon, China) according to the manufacturer’s instructions. GAPDH was used as the internal control. Three independent experiments were conducted at the same conditions.

### Kaplan-meier plotter

We used the Kaplan–Meier survival curve drawing website based on the TCGA database (http://kmplot.com/analysis/) to explore the relationship between the expression of FOXPs and the disease-free survival rate of THCA.

### UALCAN and TISIDB

An easy-to-use interactive portal for in-depth analysis of TCGA gene expression data. UALCAN (http://ualcan.path.uab.edu/index.html) uses TCGA Level 3 RNA-seq and clinical data from 31 cancer types. We used this database to analyze the relationship between FOXPs expression and lymph node metastasis. TISIDB is a comprehensive repository portal for tumor-immune system interactions. We used it to determine the Spearman correlations between FOXPs expression and 28 type of TIICs across human cancers.

### Statistical analysis

Data are expressed as the means ± SD. Analysis of variance (ANOVA) and Student’s t-test were chosen for comparison among groups. The Mann-Whitney U-test was applied in tumor volume’s comparison. Categorical data were evaluated with the chi-square test or Fisher’s exact test. P-values less than 0.05 were considered significant. Statistical analyses were processed using SPSS 22.0 (IBM, USA).

## Result

### Expression of FOXPs in different human cancers

**Figure 1:**
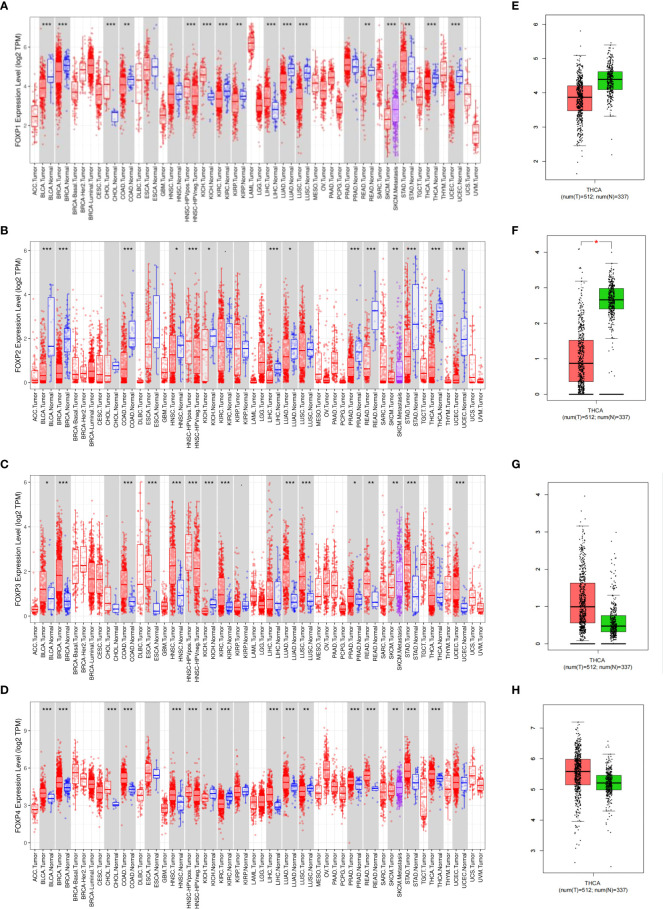
Expression of FOXPs in different human cancers. **(A)** Expression of FOXP1 in different tumor types in TCGA database was detected by TIMER. **(B)** Expression of FOXP2 in different tumor types in TCGA database was detected by TIMER. **(C)** Expression of FOXP3 in different tumor types in TCGA database was detected by TIMER. **(D)** Expression of FOXP4 in different tumor types in TCGA database was detected by TIMER. **E,** GEPIA was used to verify the difference of FOXP1 expression between thyroid cancer tissues and normal thyroid tissues in TCGA database. **(F)** GEPIA was used to verify the difference of FOXP2 expression between thyroid cancer tissues and normal thyroid tissues in TCGA database. **(G)** GEPIA was used to verify the difference of FOXP3 expression between thyroid cancer tissues and normal thyroid tissues in TCGA database. **(H)** GEPIA was used to verify the difference of FOXP4 expression between thyroid cancer tissues and normal thyroid tissues in TCGA database. *p < 0.05; **p < 0.01; ***p < 0.001.

To explore the role of FOXP1, FOXP2, FOXP3, and FOXP4 in different human cancers, TIMER and GEPIA were firstly employed to compare expression difference between normal and cancer tissues. Besides expression gap between cancer and normal tissues noticed in **Figures 1A–D**, different member of FOXPs showed divergence in expression in various cancer tissues. In a number of cancer types, including THCA, we observed that lower FOXP1, FOXP2 and higher FOXP3, FOXP4 levels in cancer tissues when compared with matched normal tissue. GEPIA database also confirmed this result that FOXP1 and FOXP2 were notably decreased in THCA, while FOXP3 and FOXP4 could be readily detected in THCA (**Figures 1E–G**).

### Expression of FOXP2 in THCA

**Figure 2:**
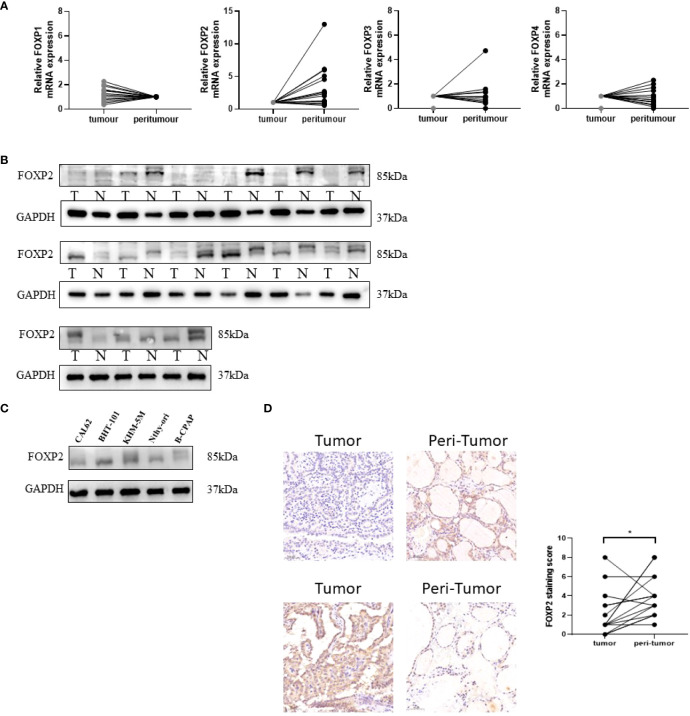
Verification of FOXP1, FOXP2, FOXP3, and FOXP4 expressions in THCA by Real Time-PCR and Western blot. **(A)** The average relative mRNA expression of FOXP1, FOXP2, FOXP3, and FOXP4 by - Real Time -PCR in DTC (tumor vs peritumor) tissues. **(B)** FOXP2 expression level in 15 paired DTC tissues detected by Western blot. **(C)** FOXP2 expression level was detected by Western blot in four thyroid cancer and normal thyroid cell lines. Each experiment is representative of three independent experiments. **(D)** 15 pairs of FOXP2 immunohistochemical staining of DTC and normal tissues, the expression of FOXP2 in cancer tissue was significantly lower than that in adjacent tissue, p=0.029(*) “*” represents comparing with the control.

Evidenced by quantitative Real Time-PCR, only FOXP2 mRNA showed the significantly down-regulation in tumor in 15 matched DTC and adjacent normal tissues (**Figure 2A**). The other members failed to demonstrate remarkable differences. In protein level, significant low expression of FOXP2 in DTC tissues was verified by Western blot in clinical samples (**Figure 2B**). A panel of cell lines including CAL62, BHT-101, KHM-5M, BCPAP and normal thyroid cell Nthy-Ori were also examined. As expected, there was a decrease in FOXP2 protein observed in a majority of THCA cell lines. FOXP2 expression in CAL62 and BCAPAP was significantly decreased, whereas, FOXP2 in BHT-101 and KHM-5M was increased than that in normal thyroid cell line Nthy-Ori.

### Prognostic potential of FOXP2 in THCA

**Figure 3:**
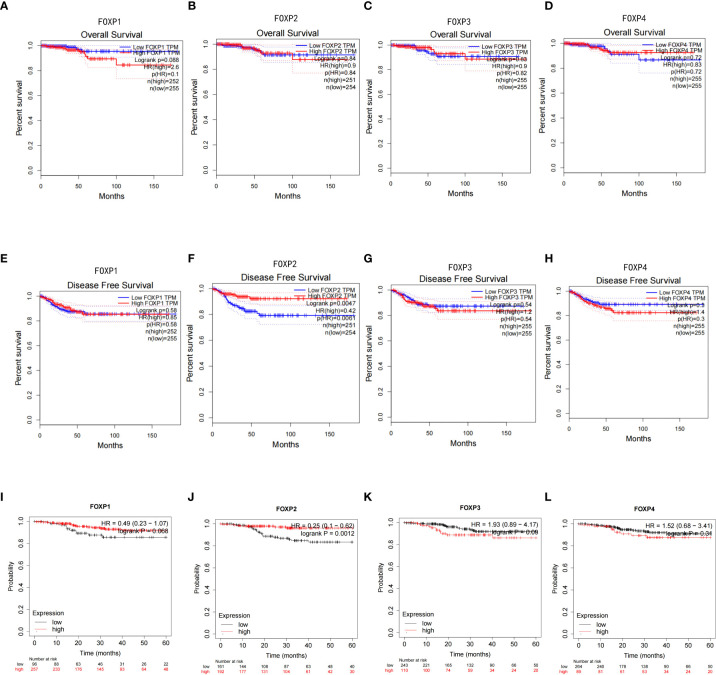
Kaplan-Meier survival curve in THCA based on the expression of FOXPs. **(A–D)** Expressions of FOXP1, FOXP2, FOXP3, and FOXP4 obtained by GEPIA on the OS survival curve of THCA. **(E–H)** Expressions of FOXP1, FOXP2, FOXP3, and FOXP4 obtained by GEPIA on the DFS survival curve of THCA. **(I–L)** Expressions of FOXP1, FOXP2, FOXP3, and FOXP4 obtained by Kaplan-Meier Plotter on the DFS survival curve of THCA.

GEPIA and Kaplan-Meier Plotter platform were then employed to explore the possibility of FOXPs as prognostic indicator for THCA. Considering the survival rate and long follow-up time, the routine criteria for prognosis determination may not be appropriate for THCA. The ability to estimate the risk of disease recurrence may be a more meaningful outcome than the risk of disease-specific death. Therefore, overall survival (OS) and disease-free survival (DFS) was evaluated here. The results showed that FOXP1, FOXP3, and FOXP4 showed no apparent effects on OS and DFS (**Figures 3A–D**). As to FOXP2, the lower expression is closely related to the shorter DFS (**Figure 3E–H**). These results proposed that down-regulation of FOXP2 may be associated with the recurrence of THCA.

**Figure 4:**
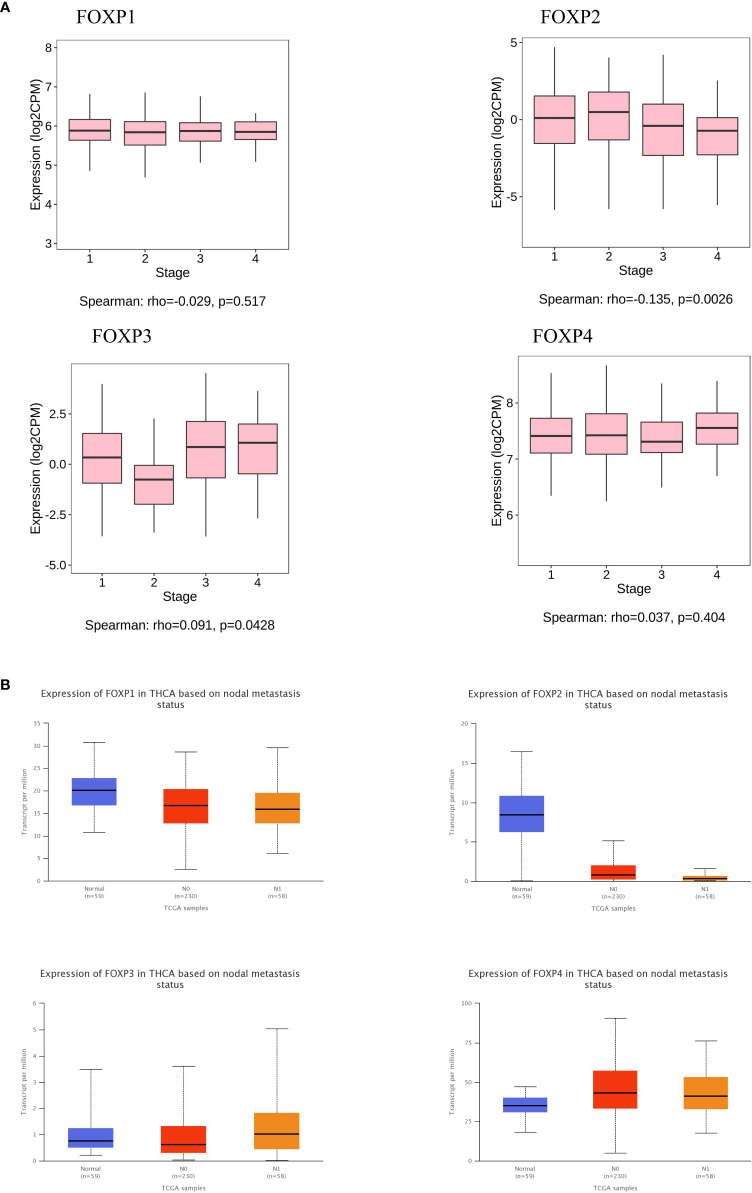
The relationship between FOXPs expression and lymph node metastasis and tumor stage. **(A)** The relationship between FOXPs expression and tumor lymph node metastasis. **(B)** The relationship between FOXPs expression and tumor stage.

Furthermore, TISIBD and UACLCAN were employed to explore FOXPs levels on lymph node metastasis and tumor staging. As shown in **Figures 4A, B**, FOXP2 has a significant influence and the down-regulation of FOXP2 could promote lymph node metastasis. Consequently, FOXP2 is closely related to tumor staging. Thus, the lower expression of FOXP2, the higher the tumor stage.

### Correlation of FOXPs and TIICs in THCA

**Figure 5:**
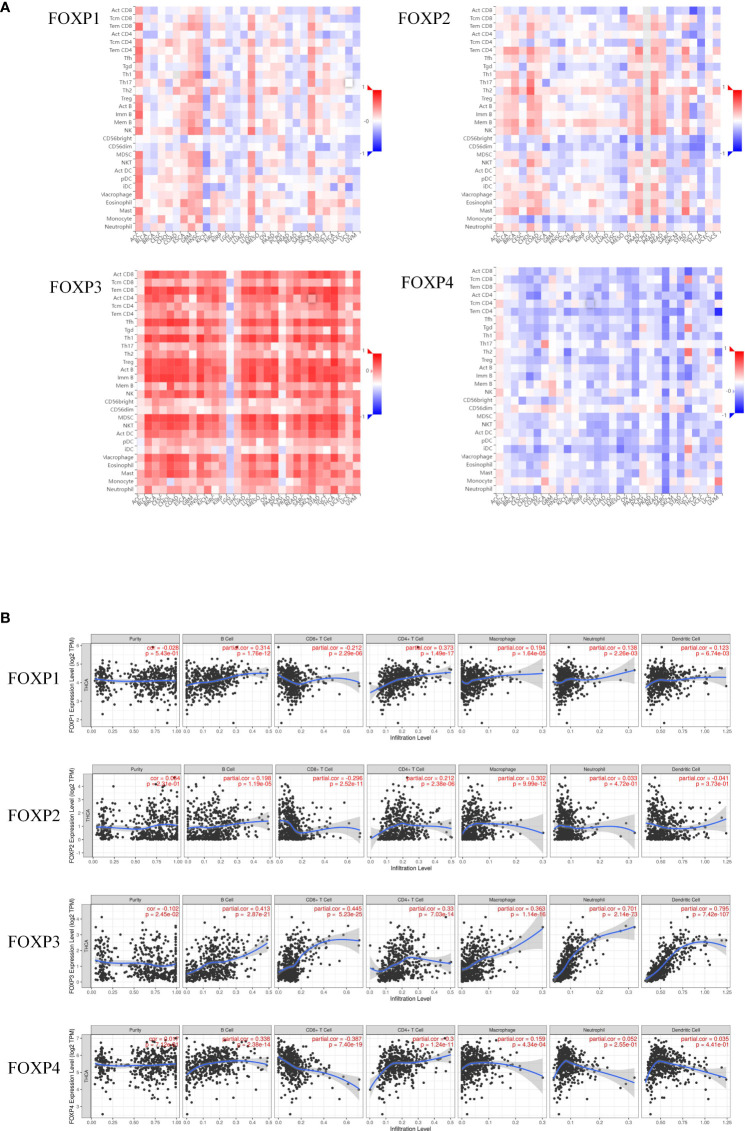
Correlation between the expression of FOXP1, FOXP2, FOXP3, and FOXP4 and the level of immune infiltration in THCA. **(A)** Expression of FOXPs with TIICs in different cancer types. **(B)** Expression of FOXP1 was significantly positively correlated with the infiltration level of B cells, macrophages, neutrophils and dendritic cells in THCA, and negatively correlated with CD8+ T cells in THCA. Expression of FOXP2 was significantly positively correlated with the infiltration level of B cells and macrophages in THCA, and negatively correlated with CD8+ T cells in THCA. Expression of FOXP3 was significantly positively correlated with the infiltration level of B cells, CD8+ T cells, macrophages, neutrophils and dendritic cells in THCA. Expression of FOXP4 was significantly positively correlated with the infiltration level of B cells and macrophages in THCA, and negatively correlated with CD8+ T cells in THCA.

TISIBD was employed to analyze the relationship between FOXP1, FOXP2, FOXP3, and FOXP4 and tumor-infiltrating immune cells in 28 kinds of tumor. As shown in **Figure 5A**, there was an inverse correlation between FOXP2, FOXP4 and immune invasion, whereas FOXP1 and FOXP3 were positively correlated.

**Figure 6:**
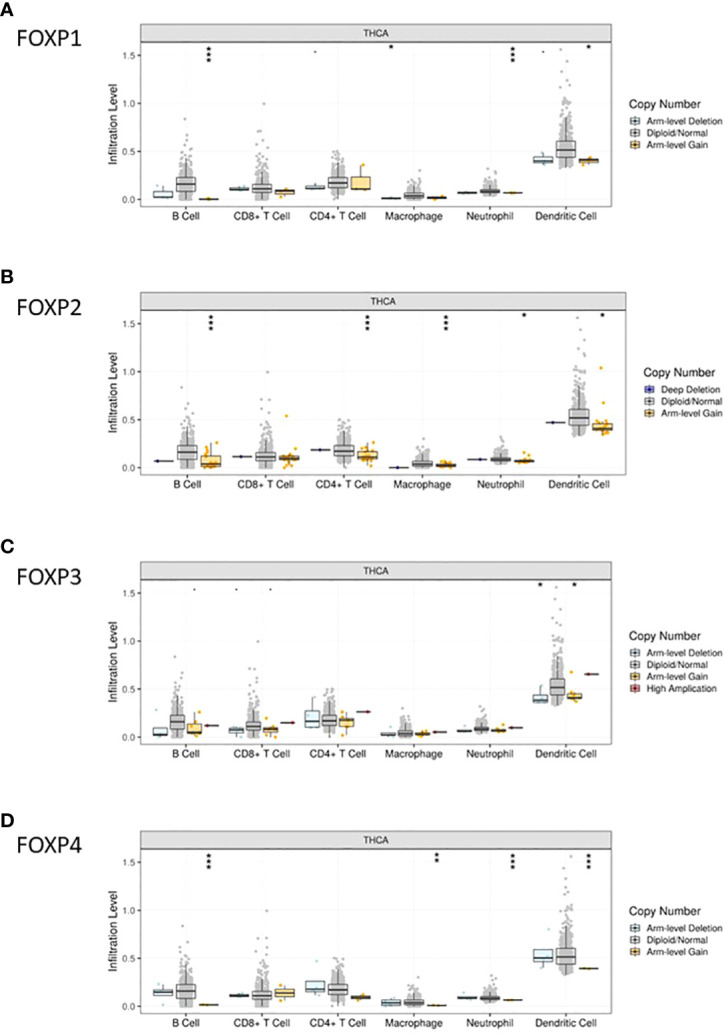
Correlation between FOXP1, FOXP 2, FOXP3, and FOXP4 expression and gene copy number. **(A–D)** Correlation between the expression levels of FOXP1, FOXP2, FOXP3, and FOXP4 and gene copy number in THCA. A, FOXP1; B, FOXP2; C, FOXP3; D, FOXP4. *p < 0.05; **p < 0.01; ***p < 0.001.

TIMER was then included to analyze the relationship between FOXPs expression and the level of immune cell infiltration quantitively. As shown in **Figure 5B**, the infiltration levels of B cells, CD4+T cells and macrophages were positively correlated with all FOXPs. There was also a significant positive correlation between the infiltration level of neutrophil, dendritic cells and FOXP1, FOXP3. A negative correlation was found between FOXP1, FOXP2, FOXP4 and the infiltration levels of CD8+T cell. In **Figure 6**, similar relationships between different gene copy numbers of FOXPs and TIICs were also found. These results strongly suggested that FOXPs play a key role in immune cell infiltration in THCA.

### Correlation between expression of FOXPs and immune cell markers sets

Based on the established relationship between FOXPs and TIICs, the involvement of FOXPs was evaluated in different immune cells markers in THCA. GEPIA analysis was initially employed and TIMER dataset was then included for standardization according to tumor purity, which could reveal more reliable results by complement of two databases. As shown in **Table 1**, **2**, the expression of FOXPs was significantly correlated with T cell (general), Monocyte, M1 Macrophage, M2 Macrophage, Dendritic cell, Th1, Th2, Tfh, Th17, and exhaustion T cell in THCA.

**Table 1:** Correlation analyses between FOXP1, FOXP2, FOXP3, FOXP4 and relate genes and markers of immune cells in TIMER.

Description	Gene markers	FOXP1				FOXP2				FOXP3				FOXP4			
		None		Purity		None		Purity		None		Purity		None		Purity	
		Cor	P	Cor	P	Cor	P	Cor	P	Cor	P	Cor	P	Cor	P	Cor	P
CD8+Tcell	CD8A	0.06	1.74E-01	0.056	2.15E-01	-0.03	5.04E-01	-0.026	5.59E-01	0.507	***	0.517	***	-0.07	1.13E-01	-0.082	7.17E-02
	CD8B	0.076	8.82E-02	0.072	1.15E-01	-0.143	**	-0.129	**	0.496	***	0.505	***	-0.032	4.75E-01	-0.036	4.34E-01
T cell(general)	CD3D	-0.099	*	-0.096	*	-0.226	***	-0.219	***	0.763	***	0.767	***	-0.231	***	-0.234	***
	CD3E	-0.059	1.86E-01	-0.056	2.18E-01	-0.178	***	-0.171	***	0.777	***	0.78	***	-0.183	***	-0.186	***
	CD2	-0.064	1.52E-01	-0.058	2.00E-01	-0.194	***	-0.191	***	0.799	***	0.799	***	-0.191	***	-0.196	***
B cell	CD19	-0.034	4.46E-01	-0.034	4.57E-01	-0.135	**	-0.137	**	0.602	***	0.595	***	-0.211	***	-0.221	***
	CD79A	-0.1	*	-0.101	*	-0.164	***	-0.162	***	0.685	***	0.687	***	-0.22	***	-0.229	***
Monocyte	CD86	-0.013	7.77E-01	-0.011	8.16E-01	-0.166	***	-0.167	***	0.798	***	0.803	***	-0.147	***	-0.151	***
	CD115CSF1R	0.07	1.15E-01	0.067	1.42E-01	-0.11	***	-0.098	**	0.689	***	0.693	***	-0.049	2.66E-01	-0.05	2.67E-01
TAM	CCL2	0.015	7.42E-01	0.022	6.30E-01	-0.104	*	-0.111	*	0.584	***	0.584	***	-0.102	*	-0.095	*
	CD68	-0.022	6.16E-01	-0.021	6.41E-01	-0.135	**	-0.139	**	0.694	***	0.688	***	-0.056	2.04E-01	-0.068	1.32E-01
	IL10	0.038	3.59E-01	0.045	3.19E-01	0.01	8.24E-01	0.021	6.43E-01	0.602	***	0.592	***	-0.106	*	-0.103	*
M1 Macrophage	INOS	0.202	***	0.201	***	0.164	***	0.175	***	0.068	1.25E-01	0.068	1.35E-01	0.195	***	0.199	***
	IRF5	-0.023	6.02E-01	-0.024	5.97E-01	-0.191	***	-0.192	***	0.563	***	0.555	***	0.179	***	0.174	***
	COX2	0.126	***	0.136	***	,,	**	-0.119	**	0.539	***	0.534	***	0.1	*	0.11	*
M2 Macrophage	CD163	0.042	3.45E-01	0.043	3.40E-01	0.015	7.33E-01	0.018	6.92E-01	0.523	***	0.519	***	-0.037	4.09E-01	-0.041	3.64E-01
	VSIG4	-0.034	4.49E-01	-0.035	4.39E-01	-0.09	*	-0.087	5.49E-02	0.601	***	0.603	***	-0.117	**	-0.118	**
	MS4A4A	-0.003	9.54E-01	-0.005	9.13E-01	-0.127	**	-0.125	**	0.638	***	0.64	***	-0.123	**	-0.133	**
Neutrophils	CD66b	0.044	3.26E-01	0.046	3.13E-01	-0.094	*	-0.089	*	0.376	***	0.376	***	-0.026	5.57E-01	-0.025	5.74E-01
	CD11b	-0.08	7.30E-02	-0.078	8.41E-02	-0.174	***	-0.179	***	0.773	***	0.774	***	-0.105	*	-0.113	*
	CCR7	-0.025	5.66E-01	-0.022	6.35E-01	-0.09	*	-0.085	6.15E-02	0.791	***	0.786	***	-0.181	***	-0.188	***
Natural KIller cell	KIR2DL1	0.07	1.16E-01	0.062	1.70E-01	0.04	3.66E-01	0.049	2.85E-01	0.04	3.66E-01	0.076	9.51E-02	-0.066	1.39E-01	-0.057	2.11E-01
	KIR2DL3	0.041	3.59E-01	0.049	2.82E-01	0.046	2.97E-01	0.059	1.94E-01	0.046	2.97E-01	0.232	***	-0.04	3.69E-01	-0.031	4.99E-01
	KIR2DL4	0.047	2.86E-01	0.06	1.83E-01	0.1	*	0.102	*	0.1	2.48E-02	0.172	***	-0.062	1.60E-01	-0.056	2.20E-01
	KIR3DL1	0.095	*	0.099	*	0.038	3.86E-01	0.049	2.80E-01	0.038	3.86E-01	0.202	***	-0.044	3.20E-01	-0.041	3.71E-01
	KIR3DL2	0.093	*	0.092	*	-0.003	9.45E-01	-0.007	8.85E-01	-0.003	9.45E-01	0.359	***	-0.092	*	-0.099	*
	KIR3DL3	-0.003	9.42E-01	-0.004	9.22E-01	-0.092	*	-0.088	5.27E-02	-0.092	3.70E-02	0.228	***	-0.0109	*	-0.118	**
	KIR2DS4	0.062	1.60E-01	0.053	2.45E-01	0.006	8.98E-01	0.016	7.22E-01	0.006	8.98E-01	0.17	***	-0.056	2.03E-01	-0.058	1.97E-01
Dendiritic cell	HLA-DPB1	-0.17	***	-0.17	***	-0.247	***	-0.246	***	0.784	***	0.789	***	-0.235	***	-0.228	***
	HLA-DQB1	-0.23	***	-0.231	***	-0.29	***	-0.29	***	0.581	***	0.581	***	-0.238	***	-0.239	***
	HLA-DRA	-0.176	***	-0.176	***	-0.205	***	-0.212	***	0.797	***	0.801	***	-0.223	***	-0.215	***
	HLA-DPA1	-0.193	***	-0.193	***	-0.245	***	-0.251	***	0.78	***	0.785	***	-0.226	***	-0.22	***
	BDCA-1(CD1C)	0	9.94E-01	0.005	9.08E-01	-0.169	***	-0.173	***	0.805	***	0.8	***	-0.06	1.85E-01	-0.059	1.86E-01
	BDCA-4(NRP1)	0.545	***	0.55	***	0.408	***	0.429	***	0.052	2.39E-01	0.03	5.13E-01	0.301	***	0.294	***
	CD11c(ITGAX)	-0.031	4.84E-01	-0.026	5.64E-01	-0.207	***	-0.211	***	0.704	***	0.699	***	-0.071	1.17E-01	-0.067	1.32E-01
Th1	T-bet(TBX21)	0.051	2.47E-01	0.059	1.94E-01	-0.053	2.36E-01	-0.046	3.15E-01	0.515	***	0.513	***	-0.113	1.06E-02	-0.111	*
	STAT4	0.068	1.25E-01	0.07	1.24E-01	-0.25	***	-0.24	***	0.692	***	0.702	***	-0.032	4.76E-01	-0.035	4.36E-01
	STAT1	0.042	3.41E-01	0.055	2.23E-01	-0.069	1.20E-01	-0.083	6.85E-02	0.66	***	0.656	***	0.133	**	0.131	**
	IFNG	-0.036	4.20E-01	-0.03	5.09E-01	-0.132	**	-0.131	**	0.6	***	0.602	***	-0.155	***	-0.157	***
	TNF	0.006	8.90E-01	0.016	7.22E-01	-0.1	*	-0.095	*	0.612	***	0.601	***	-0.101	*	-0.092	*
Th2	GATA3	0.207	***	0.205	***	-0.012	7.86E-01	-0.024	5.99E-01	0.272	***	0.258	***	0.305	***	0.298	***
	STAT6	0.322	***	0.33	***	0.157	***	0.149	9.93E-04	0.207	***	0.194	***	0.627	***	0.632	***
	STAT5A	0.14	***	0.133	***	-0.041	3.55E-01	-0.049	2.83E-01	0.428	***	0.443	***	0.312	***	0.297	***
	IL13	0.002	9.68E-01	0.012	7.98E-01	-0.112	*	-0.113	1.26E-02	0.264	***	0.268	***	-0.057	1.96E-01	-0.05	2.72E-01
Tfh	BCL6	0.287	***	0.297	***	0.239	***	0.239	***	0.319	***	0.304	***	0.273	***	0.279	***
	IL21	0.014	7.44E-01	0.01	8.21E-01	-0.012	7.83E-01	-0.015	7.37E-01	0.373	***	0.374	***	-0.139	**	-0.139	**
Th17	STAT3	0.31	***	0.324	***	0.328	***	0.323	***	0.301	***	0.285	***	0.445	***	0.448	***
	IL17A	-0.013	7.69E-01	-0.007	8.84E-01	-0.061	1.70E-01	-0.067	1.41E-01	0.333	***	0.337	***	-0.141	**	-0.135	**
Treg	FOXP3	-0.013	7.78E-01	-0.008	8.69E-01	-0.201	***	-0.21	***	1	***	-1	***	-0.083	6.03E-02	-0.086	5.72E-02
	CCR8	-0.001	9.90E-01	0.005	9.16E-01	-0.021	6.30E-01	-0.024	5.99E-01	0.793	***	0.789	***	-0.019	6.65E-01	-0.018	6.97E-01
	STAT5B	0.455	***	0.464	***	0.467	***	0.462	***	0.021	6.43E-01	0.007	8.75E-01	0.557	***	0.563	***
	TGFB1	0.373	***	0.381	***	-0.02	6.60E-01	0.002	9.73E-01	0.27	***	0.26	***	0.411	***	0.424	***
T cell exhaustion	PD-1	0.063	1.56E-01	0.066	1.47E-01	-0.105	*	-0.106	1.88E-02	0.512	***	0.53	***	-0.155	***	-0.149	***
	PDL-1	0	9.92E-01	0.007	8.73E-01	-0.076	8.81E-02	-0.073	1.09E-01	0.771	***	0.769	***	-0.146	***	-0.151	***
	CTLA4	-0.099	*	-0.094	*	-0.22	***	-0.219	***	0.855	***	0.857	***	-0.227	***	-0.23	***
	LAG3	-0.054	2.20E-01	-0.049	2.82E-01	-0.191	***	-0.182	***	0.699	***	0.709	***	-0.162	***	-0.159	***
	TIM-3	-0.011	8.00E-01	-0.009	8.44E-01	-0.158	***	-0.158	***	0.751	***	0.751	***	-0.126	***	-0.133	**
	GZMB	-0.041	3.57E-01	-0.039	3.91E-01	-0.159	***	-0.146	**	0.585	***	0.593	***	-0.244	***	-0.239	***

*p < 0.05; **p < 0.01; ***p < 0.001.

**Table 2:** Correlation analyses between FOXP1, FOXP2, FOXP3, FOXP4 and relate genes and markers of immune cells in GEPIA.

Description	Gene markers	FOXP1				FOXP2				FOXP3				FOXP4			
		Tumor		Normal		Tumor		Normal		Tumor		Normal		Tumor		Normal	
		R	P	R	P	R	P	R	P	R	P	R	P	R	P	R	P
CD8+Tcell	CD8A	0.0087	0.84	0.0052	0.97	-0.074	0.096	-0.4	**	0.37	***	0.8	***	-0.18	***	-0.35	**
	CD8B	0.11	*	-0.094	0.48	-0.032	0.46	-0.54	***	0.034	0.44	0.79	***	0.043	0.33	-0.54	***
T cell(general)	CD3D	-0.092	*	-0.11	0.41	-0.15	***	-0.54	***	0.49	***	0.66	***	-0.28	***	-0.56	***
	CD3E	-0.02	0.65	0.0019	0.99	-0.14	**	-0.48	***	0.56	***	0.9	***	-0.24	***	0.45	***
	CD2	-0.051	0.25	0.009	0.95	-0.17	***	-0.5	***	0.61	***	0.88	***	-0.27	***	-0.48	***
B cell	CD19	-0.0062	0.89	-0.032	0.81	-0.067	0.13	-0.47	***	0.35	***	0.65	***	-0.17	***	-0.47	***
	CD79A	-0.1	*	-0.091	0.49	-0.1	*	-0.52	***	0.34	***	0.72	***	-0.26	***	-0.48	***
Monocyte	CD86	0.07	0.11	0.14	0.29	-0.19	***	-0.34	**	0.7	***	0.85	***	-0.18	***	-0.32	*
	CD115(CSF1R)	0.11	**	0.18	0.17	-0.17	***	-0.26	*	0.63	***	0.89	***	-0.083	0.06	-0.28	*
TAM	CCL2	0.095	*	0.042	0.75	-0.019	0.66	-0.071	0.59	0.32	***	0.34	**	-0.077	0.083	-0.089	0.5
	CD68	0.072	0.1	0.085	0.52	-0.16	***	-0.25	0.055	0.45	***	0.72	***	-0.047	0.29	-0.14	0.28
	IL10	0.099	*	0.068	0.61	-0.045	0.31	-0.38	**	0.47	***	0.78	***	-0.13	**	-0.35	**
M1 Macrophage	INOS(NOS2)	0.15	***	-0.34	***	-0.27	***	-0.26	*	0.016	0.72	-0.037	0.78	0.23	***	-0.095	0.47
	IRF5	0.042	0.35	0.056	0.67	-0.31	***	-0.38	**	0.4	***	0.88	***	0.22	***	-0.3	*
	COX2(PTGS2)	0.23	***	0.18	0.17	-0.068	0.13	0.33	*	0.26	***	0.048	0.72	0.095	*	0.13	0.2
M2 Macrophage	CD163	0.054	0.22	0.0066	0.96	-0.14	**	-0.38	**	0.34	***	0.58	***	-0.13	**	-0.36	**
	VSIG4	0.0079	0.86	0.066	0.62	-0.17	***	-0.19	0.15	0.37	***	0.53	***	-0.15	***	-0.15	0.24
	MS4A4A	0.033	0.46	0.1	0.45	-0.16	***	-0.37	**	0.45	***	0.72	***	-0.15	***	-0.33	*
Neutrophils	CD66b(CEACAM8)	-0.012	0.79	0.22	0.091	-0.098	*	0.25	0.057	0.19	***	-0.099	0.45	-0.063	0.15	0.17	0.2
	CD11b(ITGAM)	0.024	0.6	0.21	0.11	-0.22	***	-0.21	0.1	0.62	***	0.86	***	-0.093	*	-0.15	0.24
	CCR7	0.059	0.18	0.067	0.61	-0.076	0.085	-0.42	***	0.47	***	0.83	***	-0.15	***	-0.4	**
Natural killer cell	KIR2DL1	0.049	0.27	0.026	0.84	0.0087	0.84	-0.29	**	0.14	***	0.32	*	-0.088	*	-0.25	0.06
	KIR2DL3	0.024	0.58	0.052	0.69	0.016	0.71	-0.22	0.096	0.26	***	0.28	*	-0.1	*	-0.21	0.1
	KIR2DL4	0.085	0.055	0.059	0.66	0.089	*	-0.34	**	0.2	***	0.64	***	-0.094	*	-0.23	0.085
	KIR3DL1	0.084	0.056	0.024	0.86	0.052	0.24	-0.33	*	0.25	***	0.49	***	-0.07	0.082	-0.28	*
	KIR3DL2	0.079	0.072	0.072	0.59	-0.006	0.89	-0.36	**	0.28	***	0.62	***	-0.11	*	-0.31	*
	KIR3DL3	-0.03	0.5	0.09	0.5	-0.042	0.34	-0.33	*	0.15	***	0.61	***	-0.13	**	-0.31	*
	KIR2DS4	0.065	0.14	-0.012	0.93	0.015	0.73	-0.41	**	0.22	***	0.59	***	-0.11	*	-0.32	*
Dendiritic cell	HLA-DPB1	-0.14	**	-0.086	0.52	-0.23	***	-0.53	***	0.64	***	0.74	***	-0.29	***	-0.5	***
	HLA-DQB1	-0.17	***	-0.11	0.39	-0.23	***	-0.38	**	0.36	***	0.68	***	-0.22	***	-0.32	*
	HLA-DRA	-0.12	**	0.064	0.63	-0.25	***	-0.42	***	0.68	***	0.86	***	-0.27	***	-0.4	**
	HLA-DPA1	-0.14	**	0.022	0.87	-0.23	***	-0.45	***	0.61	***	0.82	***	-0.28	***	-0.42	**
	BDCA-1(CD1C)	0.073	0.097	0.021	0.88	-0.14	**	-0.41	**	0.77	***	0.84	***	-0.14	**	-0.39	**
	BDCA-4(NRP1)	0.55	***	0.7	***	0.29	***	0.64	***	0.033	0.46	0.019	0.88	0.37	***	0.67	***
	CD11c(ITGAX)	0.023	0.61	0.11	0.39	-0.19	***	-0.29	*	0.52	***	0.87	***	-0.091	*	-0.24	0.064
Th1	T-bet(TBX21)	-0.049	0.27	0.039	0.77	-0.086	0.052	-0.44	***	0.35	***	0.82	***	-0.21	***	-0.4	**
	STAT4	0.079	0.075	0.099	0.45	-0.23	***	-0.36	**	0.54	***	0.89	***	-0.043	0.33	-0.39	**
	STAT1	0.11	*	0.28	*	-0.21	***	-0.12	0.37	0.46	***	0.76	***	0.049	0.27	-0.14	0.28
	IFNG	-0.088	*	-0.018	0.89	-0.1	*	-0.4	**	0.4	***	0.74	***	-0.24	***	-0.4	**
	TNF	-0.024	0.59	-0.012	0.93	-0.1	*	-0.3	*	0.51	***	0.61	***	-0.14	**	-0.23	0.078
Th2	GATA3	0.21	***	0.042	0.75	-0.11	**	-0.089	0.5	0.036	0.41	0.41	**	0.36	***	-0.0088	0.95
	STAT6	0.48	***	0.53	***	0.035	0.43	0.47	***	0.043	0.089	0.38	**	0.66	***	0.38	**
	STAT5A	0.29	***	0.31	*	-0.16	***	-0.038	0.77	0.33	***	0.73	***	0.42	***	-0.0088	0.95
	IL13	0.081	0.067	0.059	0.66	0.026	0.58	-0.052	0.7	0.33	***	0.25	0.059	0.0057	***	-0.072	0.59
Tfh	BCL6	0.46	***	0.035	0.37	0.15	***	0.51	***	0.16	***	0.014	0.92	0.35	***	0.29	*
	IL21	-0.0053	0.9	0.099	0.46	-0.019	0.68	-0.28	*	0.28	***	0.67	***	-0.17	***	-0.19	0.16
Th17	STAT3	0.55	***	0.65	***	0.12	**	0.59	***	0.15	***	0.13	0.32	0	0.53	0.63	***
	IL17A	-0.057	0.2	0.13	0.33	-0.097	*	0.11	0.41	0.23	***	0.18	0.18	-0.13	**	-0.021	0.88
Treg	FOXP3	-0.01	0.82	0.13	0.31	-0.22	***	-0.32	*	1	***	1	***	-0.17	***	-0.25	0.056
	CCR8	0.1	*	0.3	*	-0.076	0.084	-0.11	0.4	0.83	***	0.8	***	-0.073	0.099	-0.11	0.42
	STAT5B	0.6	***	0.64	***	0.28	***	0.64	***	0.048	0.27	0.17	0.19	0.6	***	0.65	***
	TGFβ(TGFB1)	0.33	***	-0.053	0.69	-0.16	***	-0.48	***	0.16	***	0.78	***	0.39	***	-0.38	**
T cell exhaustion	PD-1(PDCD1)	-0.056	0.21	-0.05	0.71	-0.09	*	-0.5	***	0.35	***	0.81	***	-0.22	***	-0.41	**
	PDL-1(CD272)	-0.16	**	0.18	0.16	-0.25	***	0.063	0.64	0.42	***	0.41	**	-0.13	**	-0.031	0.82
	CTLA4	-0.018	0.68	-0.011	0.93	-0.18	***	-0.44	***	0.78	***	0.71	***	-0.25	***	-0.42	***
	LAG3	-0.12	**	-0.16	0.23	-0.08	0.069	-0.58	***	0.37	***	0.71	***	-0.26	***	-0.52	***
	TIM-3(HAVCR2)	0.051	0.25	0.096	0.47	-0.19	***	-0.36	**	0.61	***	0.8	***	-0.16	***	-0.34	**
	GZMB	-0.036	0.42	-0.1	0.45	-0.11	*	-0.39	**	0.54	***	0.34	**	-0.27	***	-0.44	***

*p < 0.05; **p < 0.01; ***p < 0.001.

**Figure 7:**
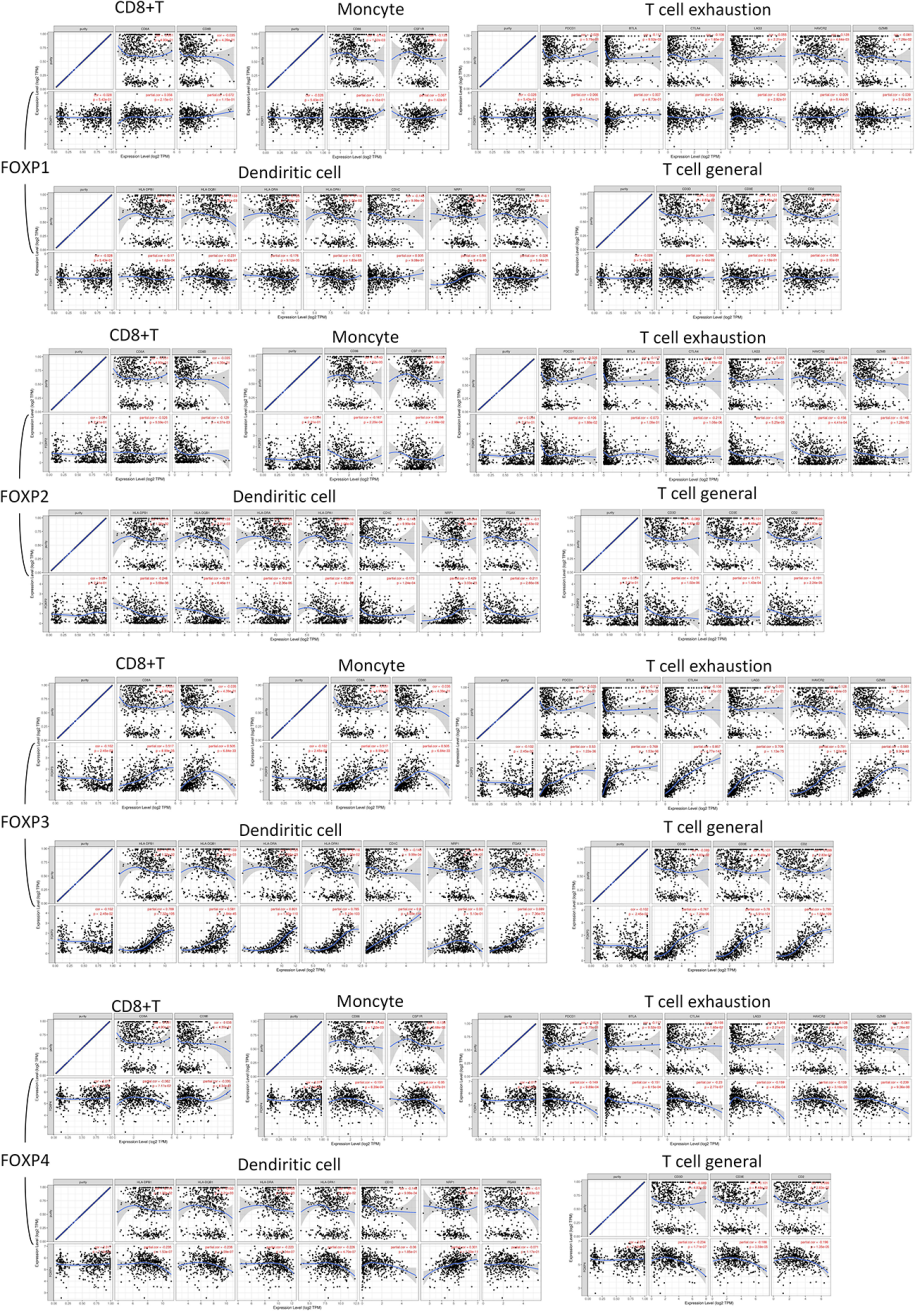
FOXP1, FOXP2, FOXP3, and FOXP4 expression correlated with TIICs. Scatterplots of correlations between FOXPs expression and gene markers of CD8+ T cell, Monocyte, T cell exhaustion, Dendritic cells, and T cell (General).

After integrating the results from two database, FOXPs showed remarkable correlations with T cells general, CD8 + T cells, Monocytes, Dendritic cells, and exhaustion T cells (**Figure 7**). In detail, FOXP2 and FOXP4 demonstrated negative correlation with most immune cell markers with positive connection observed in FOXP1 and FOXP3.

Dendritic Cell markers HLA-DPQ1, HLA-DQB1, HLA-DRA, HLA-DPA1, NRP1, and ITGAX were negatively correlated with FOXP2 significantly. It suggested that the down-regulation of FOXP2 might affect the infiltration of Dendritic cells in THCA. Moreover, there was also significant negative correlations between FOXP2 and CD86, CD115 of Monocytes, CD68 of TAM, and INOS of M1 macrophage. FOXP2 was inversely correlated with exhaustion T cell markers CTLA4, LAG3 and Treg markers FOXP3, STAT5B. Thus, FOXP2 may be involved in macrophage polarization and affect the regulation of cellular immune process in THCA.

As to FOXP1, the relationship with Dendritic cells was consistent with FOXP2. Immune markers of Th2, including GATA3, STAT6, and STAT5A, were positively correlated with FOXP1 significantly. As an immune marker, FOXP3 did show a close relation to most immune cell markers in **Figure 7**. Meanwhile, we noticed the higher expression of FOXP4 was connected to the more infiltration of Th2 cells in THCA, which meant FOXP4 might be involved in mediating Th2 invasion. FOXP4 was also positively correlated with STAT5B and TGFB1 in Treg cells. Hence, FOXP4 could facilitate tumor metastasis *via* upregulating Treg cells and diminishing CD8+T cell cytotoxicity. Taken together, more evidence supported that FOXPs were specifically associated with immune infiltrating of THCA.

## Discussion

The evolutionarily conserved family of FOX genes encompasses a large number of transcription factors involved in many developmental and differentiation processes (15, 16). As one of transcription factors, Forkhead box P (FOXP) family consists of FOXP1, FOXP2, FOXP3, and FOXP4 with similar 110 amino acid DNA-binding domain termed winged helix/forkhead domain (17). Many studies have proved FOXPs may be involved in carcinogenesis, cancer growth, tumor progression, migration, and tumor immunization (18). Previous studies suggest that the expression of FOXP factors in most human cancer can directly affect the invasion and growth of tumors, thus affecting the prognosis of patients (19, 20).

The differential expression of FOXPs between cancer and normal tissues was observed in many types of cancers. Interestingly, although FOXPs share extensive sequence similarity and appear to have very similar biological and biochemical effects, the individual family members display distinct patterns of expression and regulation. Overexpression of FOXP1 inhibits proliferation and invasion in Glioma (21). Downregulation of FOXP2 enhances tumor initiation in breast cancers as a putative tumor/metastasis suppressor (22, 23). Also, FOXP2 was downregulated in hepatocellular carcinoma (HCC) tumor tissues with poor overall survival rate and significantly promoted the invasiveness of HCC (24). FOXP3 promotes immune evasion by inhibiting Treg cell markers of cancer immune response (25). FOXP4 gene was closely associated with prostate cancer risk and is suggested a poor prognostic factor in colorectal cancer and osteosarcoma (26, 27). Detailed functional and mechanistic studies have recognized the role of FOXP factors in many cancer types such as breast cancer, osteosarcoma, and prostate cancer (28, 29). In this study, a variety of bioinformatics tools were employed to explore the effect of FOXPs on clinical indicators and immune invasion of THCA. We found that among 4 members, only low expression of FOXP2 is strongly associated with shorter DFS, higher tumor stage, and more lymph node metastases. These findings suggested that FOXPs may be a specific diagnostic and prognostic marker in cancer and targeting FOXP2 may be a potential therapeutic option for THCA.

We have shown that patients with down-regulated FOXP2 have a higher probability of tumor recurrence. Immune cell infiltration has been reported to be closely related to the progression of THCA (30, 31). Considering the possible tumor specificity of FOXPs and the significant impact of FOXP2 on the prognosis of THCA, we focused on the link between FOXP2 level and TIICs. The inseparable associations between immune cell infiltration and FOXP2 expression was firstly identified, including B cells, CD4+ T cells, CD8+ T cells, DCs, and macrophages. Based on the role of Dendritic cells in tumor immunity (32), reduced expression of Dendritic cells by FOXP2 down-regulation may promote the occurrence of tumor immune escape. FOXP2 may also increase the invasiveness of THCA and affect the prognosis by affecting infiltrating Tregs cells, due to the correlation between the invasion of Tregs cells in tumors and the aggressiveness of THCA (33, 34). Exhausted T cells, as a type of functionally limited T cells, can promote tumor development through up-regulated inhibitory receptors (35). FOXP2 expression correlates with genetic markers of exhausted T cells in THCA. Taken together, FOXP2 may promote THCA invasion and recurrence by influencing tumor microenvironment and affecting the associated immune infiltrating cells.

Our findings confirm that the contribution of FOXP2 on TIICS may trigger recurrence of THCA through the effect on Dendritic cells, Treg cells, and exhausted T cells. In this scenario, FOXP2 may be a new potential diagnostic and prognostic marker, and FOXP2 targeting therapy could be a new strategy for THCA.

## Conclusions

The present study indicated that FOXP2 was significantly correlated with markers of Dendritic cells, Treg cells and Exhausted T cells in THCA. As immune regulatory factor, the reduction of FOXP2 may affect tumor microenvironments and immune cells infiltration, enhance tumor immune escape, and promote recurrence. Therefore, FOXP2 could be a new potential diagnostic and prognostic marker. FOXP2 targeting therapy may be a promising strategy for THCA.

## Data availability statement

The datasets presented in this study can be found in online repositories. The names of the repository/repositories and accession number(s) can be found in the article/supplementary material.

## Ethics statement

This study was approved by Ruijin Hospital, Shanghai Jiao Tong University School of Medicine, and was also approved by Chinese Clinical Trial (ChiCTR2100043353). The patients/participants provided their written informed consent to participate in this study.

## Author contributions

The article was written by LX. ZY, QZ contributed equally to this work. WQ have provided guidance to the manuscript preparation. All authors have approved the final version of the editorial.

## Funding

This research was supported by the Nature Science Foundation of China (NSFC, 82072948).

## Acknowledgments

We thank all the authors who contributed to this topic. And thanks to the TCGA and GEO databases for providing data.

## Conflict of interest

The authors declare that the research was conducted in the absence of any commercial or financial relationships that could be construed as a potential conflict of interest.

## Publisher’s note

All claims expressed in this article are solely those of the authors and do not necessarily represent those of their affiliated organizations, or those of the publisher, the editors and the reviewers. Any product that may be evaluated in this article, or claim that may be made by its manufacturer, is not guaranteed or endorsed by the publisher.
